# A Pilot Study of the Total Cholesterol/High-Density Lipoprotein Ratio as a Prognostic Indicator of Hyperlipidemia-Related Diseases in Dogs and Cats

**DOI:** 10.3390/cimb46110722

**Published:** 2024-10-30

**Authors:** Kyuhyung Choi

**Affiliations:** 1 Department of Veterinary Pathology, College of Veterinary Medicine, Seoul National University, Seoul 08826, Republic of Korea; kyudac@snu.ac.kr or cokonut@naver.com; 2 Bundang New York Animal Hospital, Seongnam 13637, Republic of Korea

**Keywords:** hyperlipidemia, high-density lipoprotein, total cholesterol, Cushing’s disease, hypothyroidism, total cholesterol/high-density lipoprotein ratio, ApoA-1

## Abstract

In veterinary medicine, the significance of high-density lipoprotein (HDL) measurements is not as well documented as it is in humans. The HDL level can be measured in dogs as well and, through referring to the normal range, it is possible to find out what this means in relation to various endocrine diseases and hyperlipidemia diseases. The aim of this study is to measure the HDL levels in dogs with various conditions and to evaluate whether the total cholesterol (TC)-to-HDL ratio is effective as a prognostic indicator in various hyperlipidemia and endocrine diseases, which is significant since it is the first trial in dogs. Through a retrospective study design, sixteen client-owned dogs and cats visiting a local private practice were divided into three groups: five dogs without hyperlipidemia or metabolic disease (Group 1), eight dogs with Cushing’s, hypothyroidism, and gallbladder sludge (Group 2), and three cats, including one with diabetes, one with a urinary disorder, and one healthy cat (Group 3). In two dogs, the TC/HDL values were between 2 and 3; in two dogs, the values were between 3 and 4; and in two dogs, the values were between 4 and 5. In three dogs, the TC/HDL values were between 5 and 6 and in three dogs the ratio values were between 6 and 7. The other value was higher than 8. Except for two dogs that showed lower values than 3, all dogs in Group 1 and Group 2 had concurrent endocrine disease. This means that TC/HDL values can be an excellent indicator of endocrine disease in dogs as well. In cats, although it is a very small batch of samples, a high TC/HDL value of 9 points was shown in the cat that had diabetes mellitus. However, for more statistically significant results, a larger sample group for further investigation is needed.

## 1. Introduction

**Figure 1:**
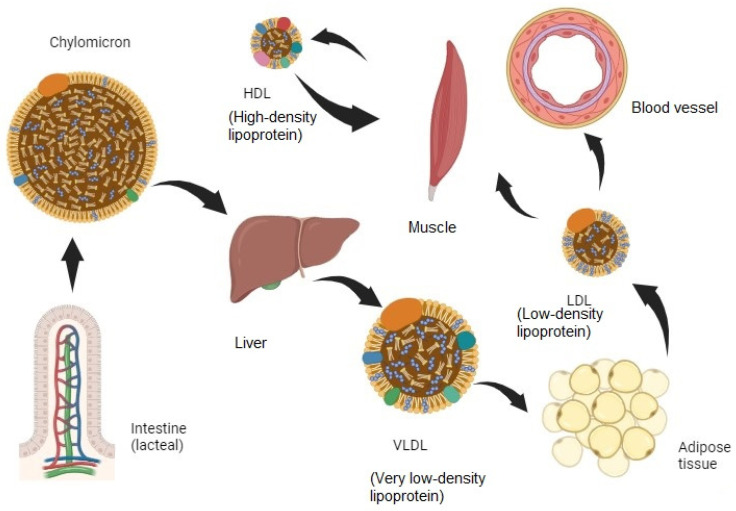
Digestion and lipid storage in animal body.

The importance of hyperlipidemia in humans has been emphasized over the years. The complications of hyperlipidemia include endocrine diseases such as diabetes and pancreatitis but fatal cardiovascular diseases such as stroke and myocardial infarction are also closely related to this condition [1,2]. Therefore, researchers have conducted studies aiming to prevent such complications through efforts to improve hyperlipidemia and, in the process, drugs to improve hyperlipidemia such as Statins and Fenofibrate [3] have been developed [4]. Lipids, which are absorbed into the body through lacteals in the small intestine after digestion, must travel through blood vessels, but since lipids are fat-soluble, not water-soluble, they cannot dissolve in the blood vessels and move on their own. Therefore, they form a lipoprotein complex and move throughout the body’s organs, changing into various forms including chylomicron, very low-density lipoprotein (VLDL), low-density lipoprotein (LDL), and high-density lipoprotein (HDL) (Figure 1). When chylomicrons are absorbed from a lacteal lymphatic vessel [5] in the small intestines, they pass through the liver to become VLDLs, then to adipose tissue to become LDLs, and then to muscles and blood vessels and other organs to condense into HDLs, and the low-density, high-volume lipids are removed.

HDL has been found to play a role in removing lipids from blood vessels which gives rise to its image as good cholesterol. As HDL levels rise, triglyceride (TG) and LDL levels decrease in relative terms, which can prevent cardiovascular diseases such as stroke and myocardial infarction. However, there has been growing awareness recently that higher HDL does not always guarantee a healthier condition and that it is better to evaluate this biomarker as a comprehensive aspect of health conditions [6].

In the veterinary field, there are relatively few studies on the role of HDL and its relationship with hyperlipidemia complications. Additionally, the frequency and importance of cardiovascular diseases such as stroke and myocardial infarction are lower than expected. One study explored the normal range of HDL in dogs [7] but HDL is not yet routinely measured in the clinical field. The findings may provide clues as to why strokes and myocardial infarctions occur less frequently in dogs than in humans. In fact, due to the shorter life spans of dogs than humans, and other critical diseases such as myxomatous mitral valve disease and lymphoma, the risk of atherosclerosis is relatively lower than in humans.

Nevertheless, arteriosclerosis and myocardial infarction due to hyperlipidemia in dogs have been reported recently [8].

In this study, the author measured HDL in dogs and cats who visited primary veterinary hospitals and examined its relationship with endocrine diseases, such as hyperlipidemia, diabetes, Cushing’s, and hypothyroidism, which is significant as it is the first trial at a local general practice. Since the number of patients visiting primary hospitals is limited, the size of the sample was determined according to statistical reference [9] and the 3R principle [10]. These are valuable data for follow-up research with a larger uniform population in referral hospitals or in universities. Also, there has been little research on HDL levels in cats. These samples of cats could provide supplementary data for follow-up research. they could also be a useful resource for follow-up research with amino acid sequence comparison.

## 2. Materials and Methods

Blood samples were collected from sixteen client-owned dogs and cats visiting Bun-dang New York animal hospital, a general practice located in South Korea (Table 1 and Table 2). The dogs and cats were selected randomly among patients aged between 3 and 14 years old, of different breeds and sexes, with or without concurrent endocrine disease who visited the hospital from May 2021 to July 2024 and were grouped into three group, five dogs without hyperlipidemia or metabolic disease (Group 1), eight dogs with Cushing’s, hypothyroidism, and gallbladder sludge (Group 2), and three cats, one with diabetes, one with a urinary disorder, and one healthy cat (Group 3). The size of the sample of dogs was determined by referring to a statistical article [9]. All blood samples were collected from the cephalic vein after physical examination (measuring body weight, BCS, and heart murmurs) in a 3 h fasted state without anesthesia or sedation and were centrifuged at 14,500 RPM for 1 min, and the sera were directly analyzed using DRI-CHEM NX500 (Fujifilm, Ratingen, Germany), a dry chemistry analyzer. Only when measuring dog HDL was the serum diluted 5 times with sterilized WFI as per the machine manual, and the other biochemistry values, including triglyceride and total cholesterol, were measured directly without dilution at room temperature. The TC/HDL and TC/TG ratios were calculated based on raw data. JASP (version, 0.19.1, the JASP team, Amsterdam, The Netherlands) was used for *t*-test data analysis (independent sample *t*-test between TC/HDL and disease) and graph creation (https://jasp-stats.org, accessed on 10 October 2024), and amino acid sequence analysis was performed using BioEdit (version, 7.2, Tom Hall, Charlotte, NC, USA, https://bioedit.software.informer.com/7.2, accessed on 10 October 2024).

## 3. Results

**Figure 2:**
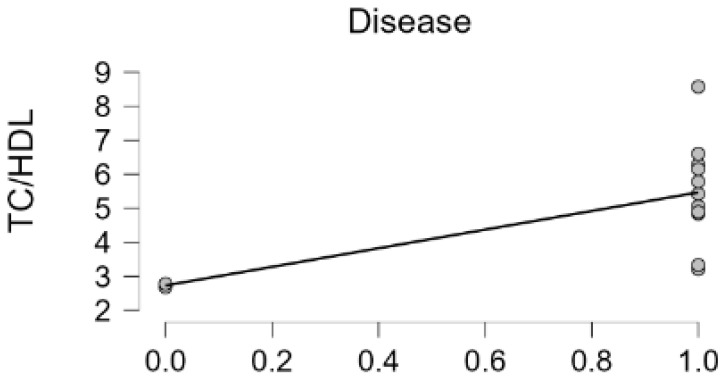
Concurrent disease (hypothyroidism, gall bladder mucocele, Cushing’s disease, hepatocellular carcinoma, and hyperlipidemia) and TC/HDL value correlation. (Pearson’s r 0.599, *p*-value 0.031).

Upon analyzing the correlation with the TC/HDL ratio by dividing the presence or absence of hyperlipidemia-related endocrine diseases, such as hypothyroidism, Cushing’s, gallbladder mucocele, and liver cancer into 1 and 0, concurrent disease and TC/HDL value showed a significant strong positive correlation (Pearson’s r 0.599, *p*-value 0.031, Figure 2). Interestingly, the dogs that had a lower TC/HDL value of less than 3 had no disease but other dogs with TC/HDL values of greater than 3 had various diseases. These data suggest that the TC/HDL cut-off value as a healthy condition biomarker could be 3, which is similar in human medicine [11] (Table 3).

In general, it is known that the higher the TC/HDL ratio in humans, the higher the likelihood of various cardiovascular diseases. Therefore, this ratio has been used as a prognostic indicator for cardiovascular diseases such as stroke and arteriosclerosis. The lower the ratio is below 5, the better the prognosis; less than 3.5 is considered good, and if it is more than 6, the risk of cardiovascular disease is high [12] (Table 3). Also, the TC/HDL ratio has been an excellent indicator for endocrine disease, such as insulin resistance diabetes [13].

The originality of this study was investigating whether the TC/HDL ratio is an effective prognostic biomarker for cardiovascular disease and endocrine disease in client-owned dogs and cats through actual measurements and it was the first trial at a general practice. There was a clear limitation in revealing a correlation between cardiovascular disease and the TC/HDL value because there was no concurrent heart disease in the samples. Nevertheless, with respect to endocrine disease related to hyperlipidemia, such as Cushing’s disease, hypothyroidism, and gallbladder mucocele, these data showed an excellent correlation between TC/HDL and concurrent diseases. In fact, in human patients with Cushing’s, a decrease in the TC/HDL ratio before and after remission can be observed, and thus, this can be used as a prognostic evaluation factor [14]. In addition, studies have shown that the incidence of gallbladder polyps is strongly related to the non-HDL/HDL ratio [15]. Also, in human hypothyroidism, the TC/HDL ratio is lowered after appropriate treatment [16]. In addition, there are research results showing that TC/HDL can be an important prognostic indicator in the case of non-alcoholic fatty liver disease, although not for liver cancer, as in our case [17]. Additionally, the TC/TG ratio was also measured to examine its potential as a prognostic evaluation factor. 

Additionally, the TC/TG value can be an indicator for small, dense LDL, which has a negative correlation in human medicine [18]. In humans, small, dense LDL is an emerging biomarker for cardiovascular disease [19,20]. It is also related to metabolic disease, including diabetes mellitus [21]. Therefore, the TC/TG value can also be negatively correlated with small, dense LDL in veterinary medicine. Pleasurably, the lowest TC/TG value was shown in the dog with hyperlipidemia and hepatocellular carcinoma #9 (Table 1). There are some studies related to LDL and hepatocellular carcinoma in human medicine [22,23], but this result needs more investigation because of the small sample and the difference between humans and dogs.

Interestingly, low HDL values were shown in dogs that had lipid metabolism-related disorders (Cushing and hypothyroidism) #6,11,12 (Table 1). Also, low HDL values were shown in diabetic cat #2 (Table 2). A low HDL level is strongly related to non-insulin-dependent diabetes mellitus (type 2) in human medicine [24] and in cats most types of diabetes mellitus are type 2 [25], so these are plausible data, although this is a case study rather than a pilot study because there is only one sample.

## 4. Discussion

**Figure 3:**
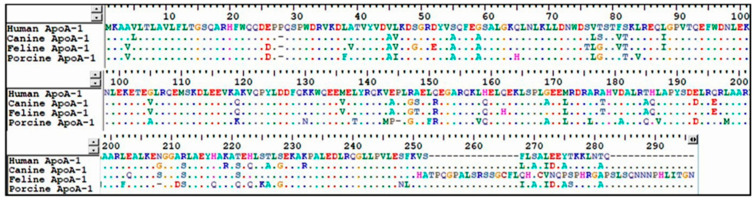
Amino acid sequence alignment of human, canine, feline, and porcine ApoA-1.

In this pilot study, the author investigated whether the TC/HDL ratio could be a biomarker for healthy condition as in humans, and the results showed that the TC/HDL value can be an excellent biomarker of lipid metabolism-related endocrine disease and its cut off value could be 3, as shown in Group 1 and Group 2. The role of HDL in the veterinary field has not yet been fully elucidated and it requires attention in additional research because of the small number of samples in this study. This study is significant as it is the first attempt to measure HDL in actual dog and cat patients using general biochemical equipment in a primary general practice rather than a laboratory. This can be applied as an annual check-up blood screening test before ultrasound or a further test for metabolic disease. Also, comparing the amino acid (Figure 3) related to HDL (ApoA-1) [26], which is identical between humans, dogs, cats, and pigs, shows that the role of HDL in cardiovascular disease may be similar among species. The reason why strokes and myocardial infarctions in dogs occur less frequently than in humans could be multifactorial [27] rather than solely genetic, and involve the environment, food, and stress [28]. Additionally, if we compare the incidence of stroke and arteriosclerosis in Schnauzers, which genetically tend to have hyperlipidemia more than other species [29], and also measure the normal range of HDL in Schnauzers, we will be able to gain a deeper understanding of the role of HDL in cardiovascular disease. Cats are more likely to develop blood clots [30] than humans, and dogs [31]. The amino acid sequence of feline ApoA-1, the related protein, is slightly different to that in other species (Figure 3). This may be related to HDL, which plays a role in transporting lipids from blood vessels to other organs [32]. To date, there has been no research on the correlation and mechanism between HDL levels and thromboembolism in cats. Further study may reveal the role of HDL in cardiovascular disease and thromboembolism in felines.

## 5. Conclusions

In this pilot study, the author randomly measured the HDL in thirteen dogs and three cats who visited a veterinary general practice and the ratio of total cholesterol to HDL turned out to be an excellent biomarker for healthy condition without diseases, with cut-off value of 3, which is similar in human medicine. This study is the first trial to reveal the correlation between HDL and hyperlipidemia and related diseases, such as hypothyroidism, Cushing’s disease, and gallbladder mucocele in dogs and cats. Further studies with larger samples at referral hospitals or universities may reveal the role of HDL in these metabolic diseases.

## Figures and Tables

**Table 1:** Signalment and concurrent disease of 13 dogs (Group 1 and 2) used in correlation and comparison study. (y: year, m: month, BCS: body condition score 1 to 9, HDL: high-density lipoprotein, TG: triglyceride, TC: total cholesterol, All blood work units: mg/dL, heart murmur: 0 to 6).

Dog#	Body Weight (kg)	BCS	Breed	Sex	Age	HDL (60~140)	TC (111~312)	TG (30~133)	TC/HDL	TC/TG	Visit Purpose	Concurrent Disease	Heart Murmur
1	3	5	Pomeranian	Spayed female	9 y	140	450	78	3.21	5.77	Annual checkup	None	0
2	23	4	Jindo	Male castrated	9 y	56	352	78	6.28	4.51	Annual checkup	None	0
3	2.2	4	Poodle	Female	9 m	64	172	218	2.68	0.79	Achilles tendon repair surgery	None	0
4	3.65	5	Maltese	Spayed female	3 y 7 m	80	223	19	2.78	11.7	Right patella luxation surgery	None	0
5	4.2	5.5	Poodle	Spayed female	7 y 7 m	56	369	104	6.6	3.54	Regular checkup	Gallbladder mucocele	0
6	3.7	4	Poodle	Male castrated	13 y	46	233	225	5.06	1.03	Regular checkup	Cushing/GERD	1
7	2.85	5	Chiwawa	Spayed female	11 y 2 m	83	450	170	5.42	2.64	Regular checkup	Cushing	0
8	5.15	5.5	Maltese	Male castrated	12 y	87	290	247	3.33	1.17	Cognitive disorder treatment	Cognitive disorder/Cushing	0
9	15.3	4	Shetland Sheepdog	Male castrated	13 y 5 m	65	315	500	4.84	0.63	Cancer treatment	Liver cancer (HCC)	2
10	2.95	4	Maltese	Spayed female	14 y 9 m	92	450	500	4.89	0.9	Cognitive disorder treatment	Gallbladder mucocele/ Cognitive disorder	0
11	8.8	4.5	Jindo	Spayed female	8 y 7 m	42	243	36	5.78	6.75	Regular checkup	Hypothyroidism	0
12	3.68	6	Pomeranian	Male castrated	4 y 9 m	42	360	402	8.57	0.89	Regular checkup	Hypothyroidism	0
13	3.5	6	Pomeranian	Male castrated	5 y 5 m	26	160	170	6.15	0.94	Dental scaling	None	0

**Table 2:** Signalment and concurrent disease of 3 cats (Group 3) for supplementary data (y: year, m: month, BCS: body condition score 1 to 9, HDL: high-density lipoprotein, TG: triglyceride, TC: total cholesterol, All blood work units: mg/dL, Heart murmur: 0 to 6).

Cat #	Body Weight (kg)	BCS	Breed	Sex	Age	HDL (60~140)	TC (85~176)	TG (17~104)	TC/HDL	TC/TG	Visit Purpose	Concurrent Disease	Heart Murmur
1	8.1	9	American Shorthair	Spayed female	5 y	75	85	21	1.13	4.04	Dysuria	Dysuria	0
2	3.9	4	Korean Shorthair	Male castrated	6 y 6 m	21	202	117	9.53	1.72	Insulin treatment	Diabetes	0
3	3	4	Korean Shorthair	Spayed female	10 m	93	138	36	1.48	3.83	OHE surgery	None	0

**Table 3:** TC/HDL value rating table.

TC/HDL Value	Rating
2.7	Good
3.5	Good
4.0	Borderline High
4.7	Borderline High
5.3	High
5.9	High

## Data Availability

Data supporting this study are included within the article.
